# Identification of TFCC substructure injury in wrist MRI using computer vision: a diagnostic aid for radiologists

**DOI:** 10.1007/s00256-025-05106-x

**Published:** 2025-12-19

**Authors:** Yongqiang Chu, Xiaolong Luo, Ruimin Guo, Hongwei Ren, Yitong Li, Gang Wu, Xiaoming Li

**Affiliations:** 1 Department of Radiology, Tongji Hospital, Tongji Medical College, Huazhong University of Science and Technology, No.1095, Jiefang Road, Wuhan, 430030 Hubei Province China; 2 Department of Radiology, Tianyou Hospital Affiliated to Wuhan University of Science and Technology, Wuhan, China

**Keywords:** Artificial Intelligence, Deep Learning, Triangular Fibrocartilage Complex, Magnetic Resonance Imaging

## Abstract

**Objective:**

The purpose of this study is to develop an automated model to assist in the detection of substructural injuries of the triangular fibrocartilage complex (TFCC), thereby reducing the reliance on subjective assessment.

**Methods:**

This retrospective study utilized 330 TFCC injured patients and 273 healthy controls from two institutions, only analyzing 2821 coronal fat-saturated T2-weighted imaging slices. From 483 samples (267 injured, 216 normal), 2292 images were processed: 1834 for training, 458 for validation, with an internal test set of 209 images from 47 samples (26 injured, 21 normal). An external test set comprised 320 images from 73 samples (37 injured, 36 normal) at another institution. Radiologists segmented and classified TFCC substructures by consensus. Different YOLO versions were trained and compared, with the optimal model benchmarked against musculoskeletal (MSK) radiologists (Resident1 and Attending2).

**Results:**

Among evaluated YOLO versions, the YOLO11l model exhibited the optimal segmentation performance, with mean Dice (mDice) coefficients of 0.82 (internal test set) and 0.77 (external test set). Its classification sensitivity, specificity, and accuracy were 91.67%, 76.11%, and 83.25% on the internal test set, significantly outperforming other versions. On the external test set, corresponding values were 84.68%, 61.22%, and 71.00%, representing the best overall performance. Notably, the diagnostic performance of the YOLO11l model was non-inferior to that of Resident1 (p = 0.41) but inferior to that of Attending2 (p = 0.015).

**Conclusions:**

The YOLO11l model represents a promising approach to aiding the assessment of TFCC injuries. Compared with less experienced radiology residents, this model can provide reliable and reproducible diagnostic support.

**Supplementary Information:**

The online version contains supplementary material available at 10.1007/s00256-025-05106-x.

## Introduction

Magnetic resonance imaging (MRI) plays a pivotal role in diagnosing internal derangement of the wrist joint. However, accurate assessment of wrist ligamentous structures, particularly the triangular fibrocartilage complex (TFCC), poses diagnostic challenges owing to the its intricate anatomy and small size (1). TFCC injuries may arise from multiple causes, including traumatic, degenerative, or combined pathological processes (2). Therefore, understanding the clinical manifestations of TFCC injuries, utilizing accurate diagnostic assessments, and appropriately managing TFCC tears are critical for optimizing patient recovery. Peripheral TFCC lesions are typically managed with reparative treatment due to their abundant vascular supply and significant healing potential (3–5).

Current literature confirms MRI as the preferred imaging modality for TFCC assessment, with higher diagnostic accuracy for central abnormalities than for peripheral lesions (4, 6, 7). MRI is non-inferior to the gold standard of diagnostic arthroscopy for identification of wrist ligamentous pathology (8). However, less experienced radiologists exhibit lower accuracy in diagnosing TFCC lesions compared with their experienced counterparts, highlighting the need for an objective protocol to facilitate early diagnosis (9).

Deep learning has advanced musculoskeletal (MSK) radiology diagnostics. While an ultrasound-based artificial intelligence (AI) model has been used to predict Palmer 1B TFCC injuries (10), our study employed You Only Look Once (YOLO) models, known for rapid object detection (11). Unlike two-stage convolutional neural networks (CNNs) that separate region segmentation and classification, YOLO integrates these processes, enabling faster detection and lower computational costs. YOLOv8 and YOLOv11, with enhanced accuracy, semantic segmentation capabilities, and optimized real-time performance, are well-suited for clinical workflows requiring rapid decision-making (12). In contrast, models such as U-Net exhibit excellent performance in pixel-level segmentation (13), but rely on more complex architectures and require greater computational resources, making YOLO a more effective alternative for clinical applications requiring fast and reliable MRI image analysis. The application of YOLO models in MSK research has demonstrated significant potential, achieving high diagnostic accuracy in meniscal tear detection (14).

Inspired by researchers that integrate attention mechanisms (AM) into computer vision systems to improve performance (15–17), the YOLOv11 architecture incorporates the innovative Cross Partial Spatial Attention (C2PSA) module, which enhances feature selection through a multi-head attention mechanism (12). Building on these advances, we systematically evaluated the diagnostic efficacy of YOLOv8 and YOLOv11 architectures for TFCC assessment using single 2D images from coronal fat-suppressed T2-weighted imaging (FS-T2WI) sequences. Given the clinical significance of vascularized zones in TFCC, peripheral tears are frequently missed on imaging and often require surgical intervention (5, 18, 19). Early detection and timely management of these TFCC lesions can significantly improve repair outcomes. However, radiologists face challenges in accurately diagnosing TFCC injuries, particularly in resource-limited settings (20). Our study aims to propose an automated model for detecting, segmenting, and diagnosing TFCC injuries, providing an efficient and accurate diagnostic aid.

## Methods and materials

### Dataset

**Fig. 1:**
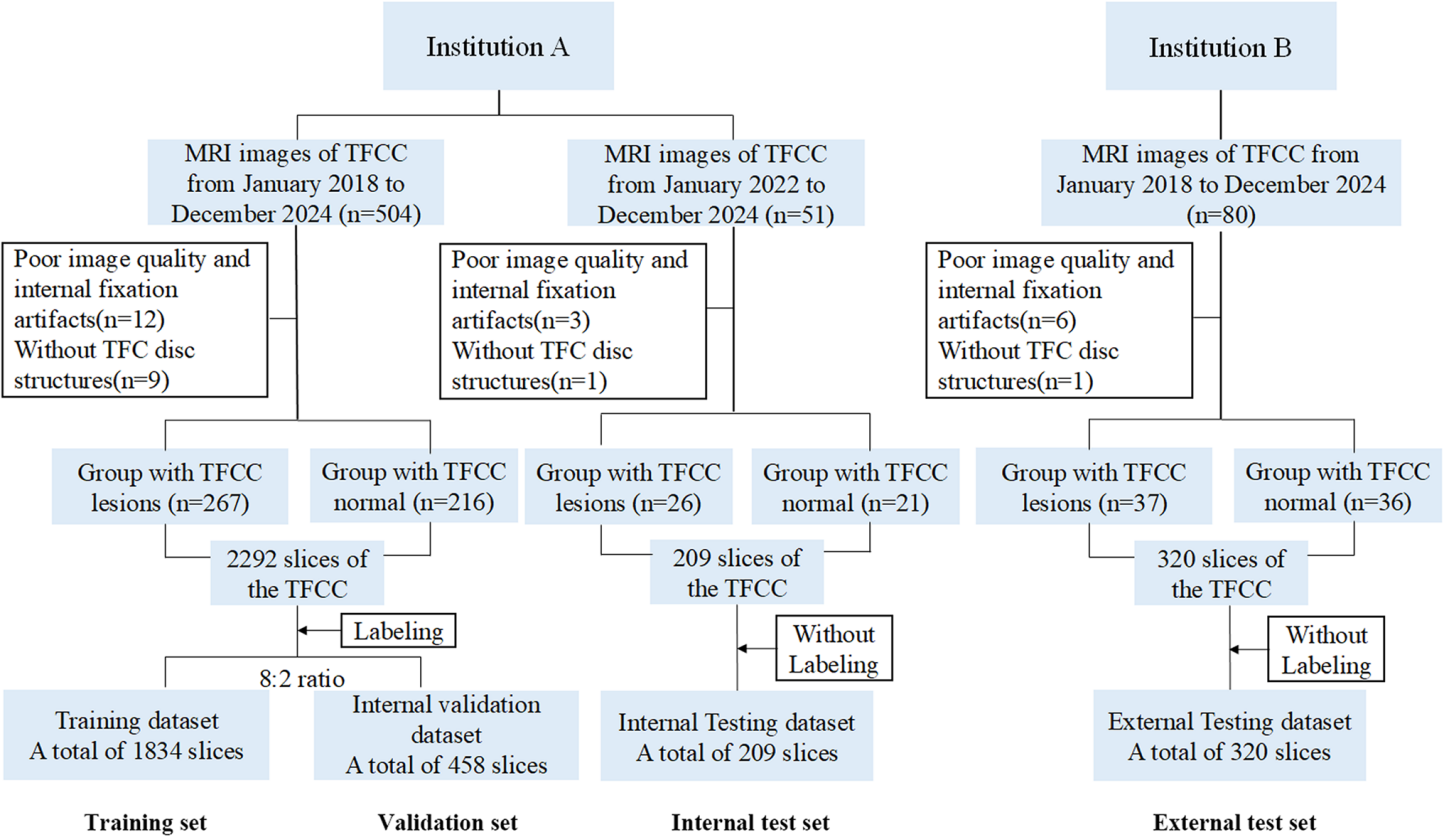
Flowchart of dataset selection process

This study was approved by the Institutional Review Board (IRB NO.TJ-IRB202501013), and as it was a retrospective study, informed patient consent was waived. Wrist MRI data were collected from two different institutions, including 530 cases from institution A and 73 cases from institution B, both of which included images of patients with TFCC injuries and healthy controls. The inclusion criteria for the patient group were: (a) clinical symptoms or medical history of acute or chronic ulnar wrist pain, (b) injury classification encompassing all Type I (traumatic) lesions and Type IIA, IIB, and IIC lesions, (C) age ≥ 18 years, and (d) availability of coronal FS-T2WI sequences. The inclusion criteria for healthy subjects were (a) presence of wrist discomfort but no abnormalities identified on MRI, and (b) cases with normal physical examination. The exclusion criteria were (a) lacking TFCC disc structures, and (b) images with poor quality and internal fixation artifacts. The process of dataset selection is shown in Fig. 1.

Data splitting was performed at the patient level to avoid data leakage, ensuring all images from the same patient were assigned to the same subset. Demographic data of patients with TFCC injuries and healthy subjects across the three datasets (training and validation, internal test, and external test) are shown in Table 1. Of the 2527 slices from institution A, 209 unlabeled slices were used as internal test sets, while the remaining 2292 labeled slices were split into training and validation sets in an 8:2 ratio, resulting in 1834 and 458 slices, respectively. Additionally, 320 unlabeled slices from institution B formed the external test set.

**Table 1.** Demographic and Gender Distribution in the control and patient groups

	Training + Validation Dataset	Internal Testing Dataset	External Testing Dataset
**Patient group**			
Patients (n)	267	26	37
TFCC lesions (n)	275	28	39
Age (years, mean ± SD)	53.5 ± 13.8	51.2 ± 15.3	51.7 ± 14.3
Male (n)	130	14	17
Female (n)	137	12	20
**Control group**			
Healthy Individuals (n)	216	21	36
Healthy TFCC (n)	220	23	37
Age (years, mean ± SD)	45.8 ± 14.1	43.2 ± 11.3	43.5 ± 12.3
Male (n)	103	12	18
Female (n)	113	9	18

Based on clinical records and Palmer classification, TFCC injuries were categorized into traumatic and degenerative types. When evaluating structures, radiologists were required to label both normal cases (confirming no lesions) and abnormal cases (traumatic or degenerative lesions). All data was segmented by a radiologist with 7 years of experience (Y.C.), and the segmentation results were reviewed and verified by a radiologist with 15 years of experience (G.W.). The classification of all segments was carried out by two radiologists (H.R. with 15 years of experience, Y.L. with 8 years of experience). In cases of classification discrepancies, a radiologist with 30 years of experience (X.L.) was consulted for a final decision. Consensus interpretation was adopted to improve the reliability of ground truth annotation.

### Data processing

#### Slice conversion and selection

We selected the FS-T2WI sequence as the input for the YOLO model due to its diagnostic advantages, specifically its high sensitivity to soft tissue and fluid signal characteristics, provide additional value for detecting peri-lesional edema and inflammatory changes associated with TFCC injuries (21–24).

All images were acquired from 3 Tesla (T) clinical scanners (Institution A: Signa Pioneer, GE Healthcare, USA; Magnetom Skyra, Siemens Healthcare, Germany; uMR770, uMR780, uMR790, Shanghai United Imaging Healthcare, China; Institution B: Ingenia 3.0 T, Philips Healthcare, Netherlands).

Prior to model training, comprehensive data preprocessing was performed. MRI images in DICOM format were exported from the Picture Archiving and Communication System (PACS) and converted to PNG format (640 × 640 pixel) using Pydicom, OpenCV, and Matplotlib libraries. This format conversion was implemented to reduce computational load while preserving sufficient resolution for identifying critical TFCC structural features. We extracted 2D slices from the FS-T2WI sequence for processing, focusing on feature extraction from specific anatomical slices. This approach was adopted because 2D processing facilitates more efficient data annotation and accelerates model training. The methodological validity is supported by precedent studies successfully employing 2D image analysis for comparable diagnostic tasks (14, 25).

#### Image annotation and dataset splitting

Typically, the dorsal and volar radioulnar lineaments belong to the central structure. however, On the coronal FS-T2WI sequence, the volar radioulnar ligaments, ulnotriquetral ligament, and ulnolunate ligament often appearing on the same slice and difficult to distinguish clearly. To reduce annotation complexity, we classify the dorsal and volar radioulnar ligament as peripheral structures, which does not affect the recognition of target. The LabelMe tool (https://github.com/CSAILVision/LabelMeAnnotationTool) was used to segment central structures (triangular fibrocartilage disc and triangular ligament) and peripheral structures (dorsal and volar distal radioulnar ligaments, ulnotriquetral ligament, and ulnolunate ligament) in images from Institution A, categorizing them into four classes: abnormal central structures, normal central structures, abnormal peripheral structures, and normal peripheral structures. Segmentation was performed using pixel-wise annotation techniques with polygonal contour delineation for precise boundary definition. The types and quantities of slices across the four datasets (training, validation, internal test, and external test) are shown in Table 2.

**Table 2.** Slices and group of FS-T2WI sequence across different datasets

lesion category group	Training Dataset (slices)			Validation Dataset (slices)			Internal Testing Dataset (slices)			External Testing Dataset (slices)		
	abnormal group	normal group		abnormal group	normal group		abnormal group	normal group		abnormal group	normal group	
Central structures (TFC disc, triangular ligament) (n)	511		294	72		104	48		30	61		31
Peripheral structures (DRUL, VRUL, UTL, and ULL) (n)	337		692	131		151	48		83	63		165

TFC: triangular fibrocartilage, DRUL and VRUL: dorsal and volar distal radioulnar ligaments, UTL: ulnotriquetral ligament, ULL: ulnolunate ligament

#### Model developing

**Fig. 2:**
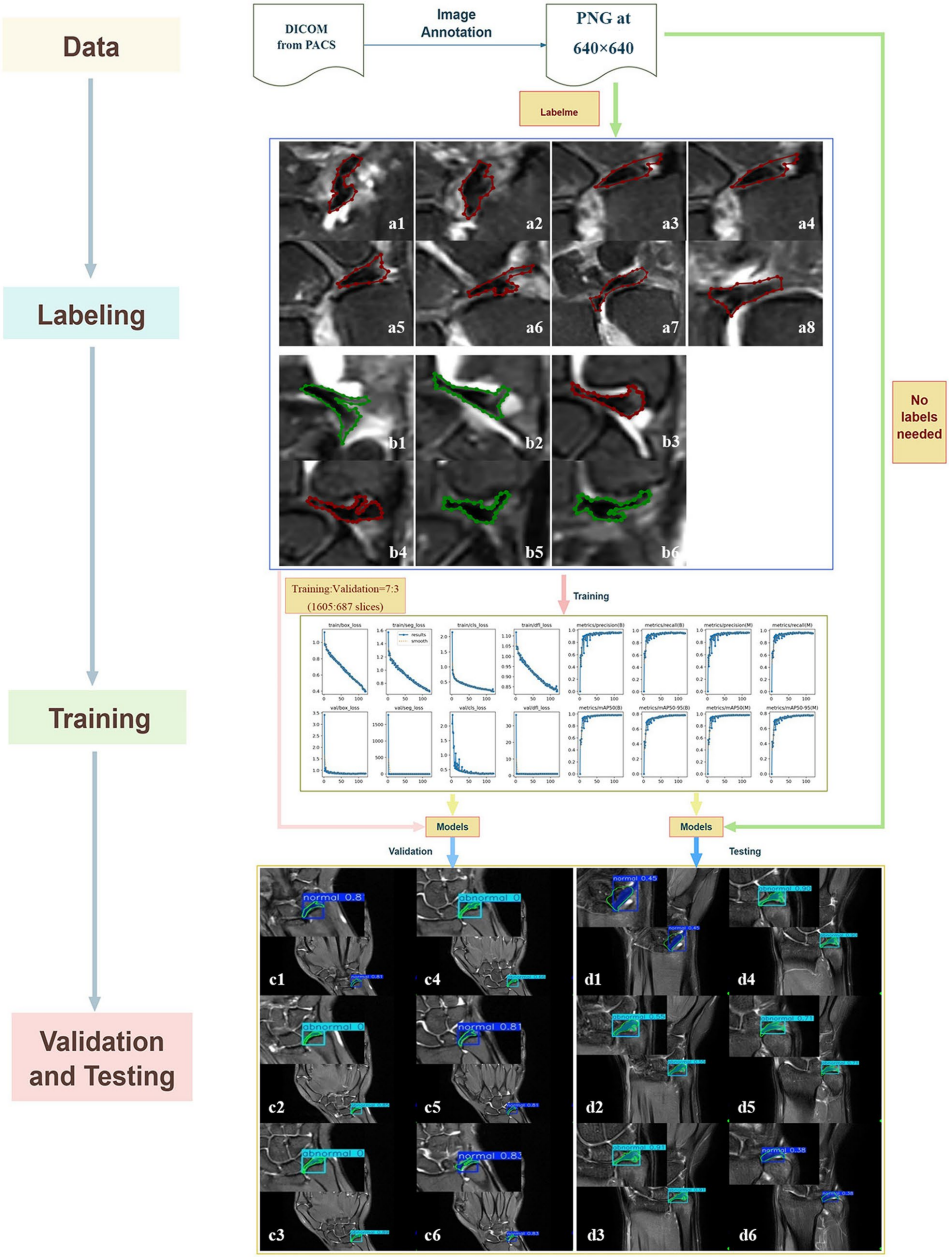
Workflow in MRI Diagnosis of TFCC lesion. A patient (a1-a8) with TFCC injury, where the central structures (a3, a4) and peripheral structures (a1, a2, a5-a8) are marked with red polygons indicating structural damage. Another patient (b1-b6) with TFCC injury, where red polygons are used to indicate damage to the central structures (b3, b4), and green polygons are used to indicate normal peripheral structures (b1, b2, b5, b6). After model validation and testing, validation set examples (c1-c6) and test set examples (d1-d6) were obtained. The generated detection boxes, predicted segmentation masks, predicted categories, confidence levels, and polygon annotations of the original segmentation can be seen in the figure. Most of the predicted segmentation mask ranges have high overlap accuracy with the polygon annotations of the original segmentation, while some overlap accuracy is average (such as c2, c6), and a few overlaps accuracy is poor (d1)

The development and training environment was set up using Python 3.11.4 (https://www.python.org/) for all deep learning workflows. The YOLOv8 and YOLOv11 frameworks were implemented via the Ultralytics library (version 8.3.181; https://www.ultralytics.com/), which was built on PyTorch 2.0.0 (https://pytorch.org/). All models were trained with standardized parameters: input image size of 640 × 640 pixels, batch size of 16, and initial learning rate (lr0) of 0.01. To enhance model robustness, data augmentation techniques were applied during training, including random horizontal flipping, random vertical flipping, random rotation (± 15°), and random brightness/contrast adjustments (± 20%). Each model was trained for 200 epochs on an NVIDIA A10 GPU with 24 GB of VRAM. The training process and output generation are depicted in Fig. 2.

#### Deep-learning model and network architectures

**Fig. 3:**
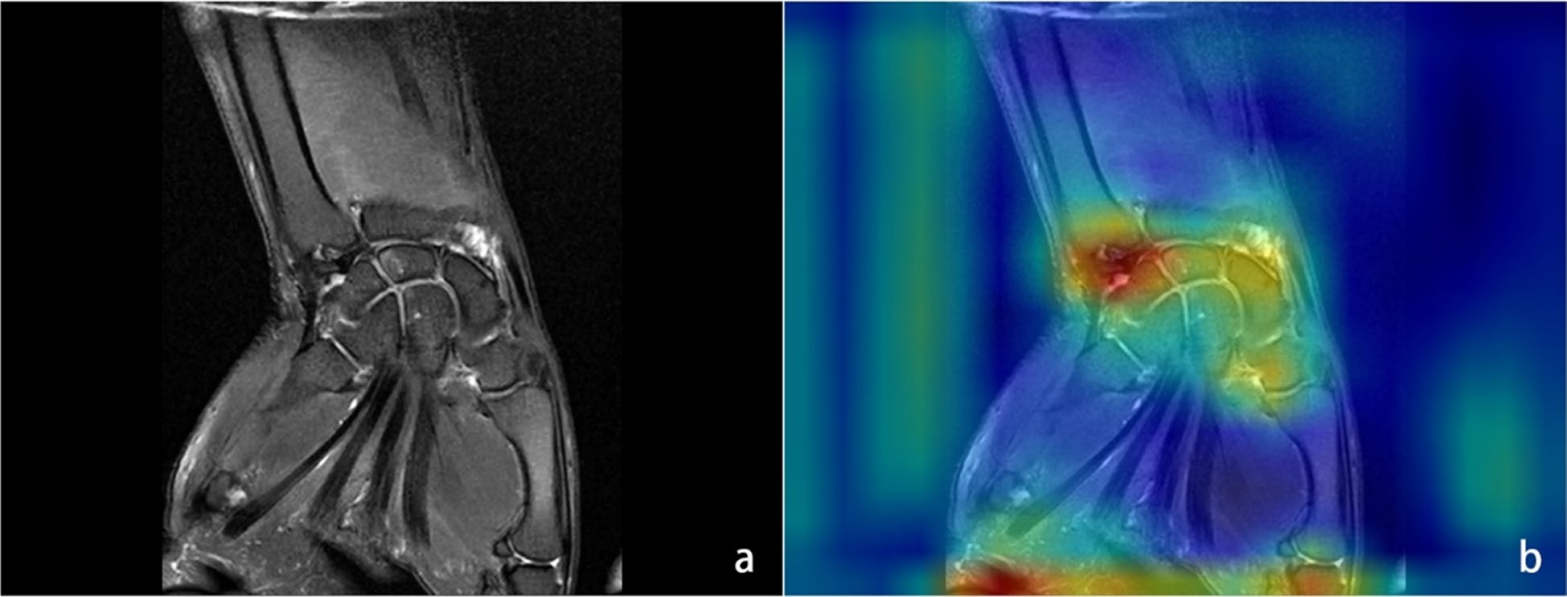
The heatmap shows the impact of the YOLO11m model on the TFCC focus. (**a**) FS-T2WI, (**b**) heatmap: After deep learning through convolutional layers, attention shows strong focus on TFCC

**Fig. 4:**
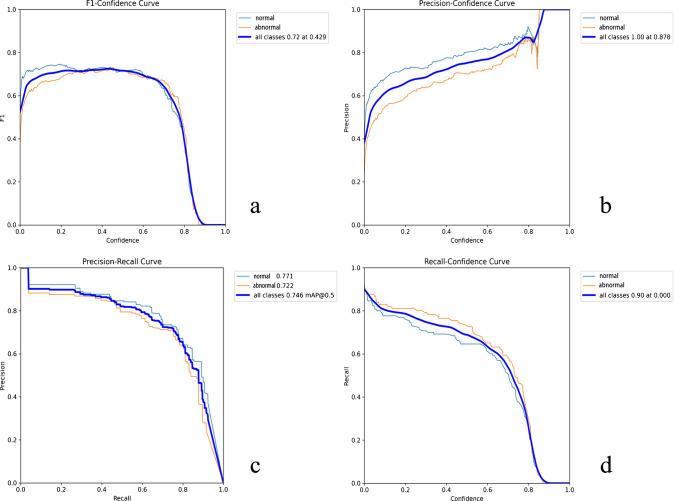
Classification performance of YOLO11l model after training and validation. Respectively represent the F1 Score (**a**), Precision (**b**), Precision-Recall Curve (**c**), and Recall (**d**) of classification (**a**-**d**) performance. The YOLO11l model performs better than other YOLO models on the validation set, and there are statistically significant differences in sensitivity, specificity, and accuracy compared to some models

YOLOv8 and YOLOv11 differ in key architectural features relevant to our clinical task. YOLOv8 uses optimized gradient routing and pooling modules, with structure adjustments enabling anchor-free detection for more efficient processing (15). YOLOv11 builds on this with C3K2 modules for early feature extraction and incorporates attention mechanisms (C2PSA) after pooling layers, strengthening its ability to select and integrate critical features in complex medical images (12). Both models maintain the speed required for clinical workflows. Figure 3 shows the AM heatmap of the model, and the classification performance of YOLO11l is depicted in Fig. 4.

#### Model evaluation

The YOLO implementation (https://github.com/ultralytics/ultralytics) automatically generates per-class false negative rates and correct diagnosis rates, yielding precision metrics, confidence levels, F1-scores, and confidence relationship plots.

To rigorously evaluate model performance, we calculated overall (injury and normal) accuracy, sensitivity, specificity, and other metrics that distinguish between lesion and healthy segmentations, as well as between central and peripheral structures. We compared the performance of 10 models, including segmentation accuracy (Dice coefficient) and classification metrics (F1 score, precision, precision-recall curve, recall) (Supplementary Fig. 1–4). This approach enables standardized statistical comparisons, providing insights into the relative advantages of different models. Additionally, we also evaluated the performance differences across different YOLO versions (Table 3) for sensitivity, specificity, and accuracy.

**Table 3.** Overall classification diagnostic performance of YOLOv8 and YOLOv11 models on Testing Datasets

	Internal Testing Dataset				External Testing Dataset			
Model	Sensitivity	Specificity	Accuracy	P	Sensitivity	Specificity	Accuracy	P
				0.49*				0.08*
YOLO8n	87.50% (80.88%, 94.12%)	76.11% (68.24%, 83.97%)	81.34% (76.06%, 86.62%)		57.26% (47.85%, 66.28%)	63.27% (56.13%, 69.95%)	60.83% (55.02%, 66.38%)	
YOLO8s	86.46% (79.61%, 93.30%)	75.22% (67.26%, 83.18%)	80.38% (75.00%, 85.77%)		67.22% (57.98%, 75.54%)	62.76% (55.61%, 69.46%)	65.17% (59.38%, 70.63%)	
YOLO8m	88.54% (82.17%, 94.91%)	76.99% (69.23%, 84.75%)	82.30% (77.12%, 87.47%)		79.03% (70.54%, 86.02%)	59.18% (52.00%, 65.99%)	68.00% (62.28%, 73.36%)	
YOLO8l	83.33% (75.88%, 90.79%)	78.76% (71.22%, 86.30%)	80.86% (75.53%, 86.19%)		75.20% (66.45%, 82.51%)	58.16% (51.00%, 65.01%)	66.17% (60.40%, 71.60%)	
YOLO8x	77.08% (68.68%, 85.49%)	76.99% (69.23%, 84.75%)	77.03% (71.33%, 82.74%)		73.39% (64.54%, 80.91%)	50.51% (43.50%, 57.50%)	61.17% (55.36%, 66.72%)	
				0.93*				0.004*
YOLO11n	86.46% (79.61%, 93.30%)	76.99% (69.23%, 84.75%)	81.34% (76.06%, 86.62%)		59.68% (50.20%, 68.67%)	59.18% (52.00%, 65.99%)	59.33% (53.50%, 64.93%)	0.50^#^
YOLO11s	86.46% (79.61%, 93.30%)	78.76% (71.22%, 86.30%)	82.30% (77.12%, 87.47%)		67.22% (57.98%, 75.54%)	65.31% (58.26%, 71.81%)	66.17% (60.40%, 71.60%)	0.29^#^
YOLO11m	87.50% (80.88%, 94.12%)	75.22% (67.26%, 83.18%)	80.86% (75.53%, 86.19%)		89.43% (82.51%, 94.23%)	50.51% (43.50%, 57.50%)	68.00% (62.28%, 73.36%)	0.67^#^
YOLO11l	91.67% (86.14%, 97.20%)	76.11% (68.24%, 83.97%)	83.25% (78.19%, 88.32%)		84.68% (76.70%, 90.53%)	61.22% (54.08%, 67.98%)	71.00% (65.34%, 76.21%)	0.003^#^
YOLO11x	84.38% (77.11%, 91.64%)	78.76% (71.22%, 86.30%)	81.34% (76.06%, 86.62%)		75.81% (67.12%, 83.14%)	53.55% (46.50%, 60.52%)	63.67% (57.87%, 69.20%)	0.02^#^
8n vs 11n				1.00				0.60
8 s vs 11s				0.52				0.60
8 m vs 11m				0.62				0.67
8 l vs 11l				0.41				0.04^!^
8 × vs 11x				0.15				0.29

*The results of the Friedman test indicate that there is a significant difference within the YOLO11 group in the external test set

#According to the Wilcoxon signed-rank test with Bonferroni correction within the YOLO11 group, p < 0.005 indicates a significant difference. Only 11n vs 11 s, 11n vs 11 m, 11n vs 11 l, 11 m vs 11 l, 11 l vs 11x are displayed here

! The Wilcoxon signed-rank test results indicate that there is a significant difference between 11l and 8 l in the external test set

#### Evaluation of model performance versus two radiologists

To evaluate models’ performance, we randomly selected 172 structural injury images and 193 structural health images from internal and external test sets for comprehensive assessment. Firstly, two radiologists (1: X.L., 2: R.G.) independently evaluated the images without knowledge of the model output, and then their results were compared with those of the YOLO11l model. Resident 1 had one year of general diagnostic experience without specialized MSK imaging training, while Attending 2 had five years of diagnostic experience with specialized MSK imaging training.

### Statistical analysis

We compared demographic characteristics, including age and gender, of the structural injury and the healthy control group across three datasets (training + validation, internal test, and external test). The Shapiro–Wilk test was used to assess the normality of age distribution, and the Levene test to assess variance homogeneity. The Mann–Whitney U test was used to compare age differences between the injury and the healthy control groups within each dataset. Gender differences were analyzed using the chi-square test. The Wilcoxon signed-rank test was used for pairwise comparisons within the same dataset. A p < 0.05 was considered statistically significant.

## Results

### Demographics and statistical comparisons

When comparing the same groups across the three datasets, no significant age differences were observed in either the injury group or the control group (p > 0.05). However, when comparing injury group against control group within each individual dataset, significant age differences were observed in all datasets (p < 0.01), with consistently younger demographics in control populations. This age disparity may reflect the inclusion of individuals with other wrist pathologies in the control cohort.

### TFCC segmentation accuracy

We statistically analyzed the segmentation performance of 10 models. The YOLO11l model exhibited the highest segmentation accuracy for the TFCC structure in the internal test set (mDice = 0.82), which was the best among all evaluated models. We also compared the group segmentation performance of 10 models (Supplementary Table 1). In the internal test set, no significant statistical differences were observed between the five YOLOv11 models (11n, 11 s, 11 m, 11 l, 11x), between 8 and 11n, 8 s and 11 s, 8 m and 11 m, 8 l and 11 l, or 8 × and 11x. However, a significant difference was observed between 8n and 8 m (p = 0.0048).

In the external test set, YOLO11l still showed the highest segmentation accuracy (mDice = 0.77). We also compared the group segmentation performance of 10 models (Supplementary Table 1). There are significant statistical differences between multiple pairs, such as 8n and 8 m, 8 m and 8 l, 11n and 11 m, 11 m and 11 l, 8 m and 11 m (p < 0.001, p < 0.001, p < 0.001, p < 0.001, p = 0.002). However, there is no significant statistical difference between 8 and 11 l (p = 0.12).

### TFCC lesion detection rate

In the internal test dataset, no significant statistical differences were observed between all models. Table 3 summarized a comprehensive summary of the performance of the model on the internal test set. In addition, we also presented a comparison of classification metrics for 10 models after training and validation, including F1-Confidence curve、Precision-Confidence curve、Precision-Recall curve、Recall-Confidence curve (Supplementary Fig. 1–4). Compared with all other models, the YOLO11l model showed the best performance (Sensitivity (95% CI), Specification (95% CI), and Accuracy (95% CI) were 91.67% (86.14%, 97.20%), 76.11% (68.24%, 83.97%), and 83.25% (78.19%, 88.32%), respectively). It is worth noting that compared to YOLO8l, YOLO11l has a higher sensitivity (91.67% (86.14%, 97.20%) vs 83.33% (75.88%, 90.79%)), demonstrating superior higher performance in detecting TFCC structural damage. YOLO11l model exhibits excellent performance, highlighting its clinical utility for comprehensive evaluation of central and peripheral lesions using coronal FS-T2WI sequences.

There were significant statistical differences (p = 0.003, p = 0.04) between 11 l and 11n, 11 l and 8 l in the external test dataset. No significant statistical differences were observed among the other models. Table 3 summarized a comprehensive summary of the performance of the model on the external test set. Compared with all other models, the YOLO11l model exhibits the best performance (Sensitivity (95% CI), Specification (95% CI), and Accuracy (95% CI) of 84.68% (76.70% −90.53%), 61.22% (54.08% −67.98%), and 71.00% (65.34% −76.21%), respectively). It is worth noting that compared to the YOLOv8 architecture, YOLOv11 shows a slight improvement and higher performance in detecting TFCC structural damage. Among them, the sensitivity and accuracy of YOLO11l are significantly stronger than YOLO8l.

We demonstrated the performance of 10 models in diagnosing by region classification on the test dataset (Supplementary Table 2). As shown in Table 4, the YOLO11l model has the best overall performance in evaluating the sensitivity, specificity, and accuracy for central and peripheral structural lesions across internal and external test datasets.

**Table 4.** Regional classification diagnostic Performance of YOLO11l Models on Testing Datasets

Internal Testing Dataset	Sensitivity	central	91.67% (83.85%—99.49%)
		peripheral	91.67% (83.85%—99.49%)
	Specificity	central	83.33% (70.00%—96.67%)
		peripheral	73.49% (64.00%—82.99%)
	Accuracy	central	88.46% (81.37%—95.55%)
		peripheral	80.15% (73.32%—86.98%)
External Testing Dataset	Sensitivity	central	91.80% (84.92%—98.69%)
		peripheral	77.78% (67.51%—88.04%)
	Specificity	central	90.32% (79.92%—100.73%)
		peripheral	55.76% (48.18%—63.34%)
	Accuracy	central	91.30% (85.55%—97.06%)
		peripheral	61.84% (55.54%—68.15%)

### Diagnosis comparison with radiologists

We evaluated 200 randomly selected images from internal and external test sets using the trained YOLO11l model. Compared with Resident 1 and Attending 2, the YOLO11l model performed better than Resident 1, as shown in Table 5.

**Table 5.** Classification evaluation between YOLO11l Models and two residents on external testing dataset

	Result				Performance			
	TP	FN	TN	FP	Sensitivity (95% CI)	Specificity (95% CI)	Accuracy (95% CI)	P
YOLO11l	79	21	70	30	79.0% (71.0% ~ 87.0%)	70.0% (61.0% ~ 79.0%)	74.5% (68.5% ~ 80.5%)	
Resident1	74	26	64	36	74.0% (65.4% ~ 82.6%)	64.0% (54.6% ~ 73.4%)	69.0% (62.6% ~ 75.4%)	0.41
Attending2	87	13	80	20	87.0% (80.4% ~ 93.6%)	80.0% (72.8% ~ 87.2%)	83.5% (78.3% ~ 88.7%)	0.015*

*Using YOLO11l as a benchmark, perform Wilcoxon sign-rank test after Bonferroni correction

**Fig. 5:**
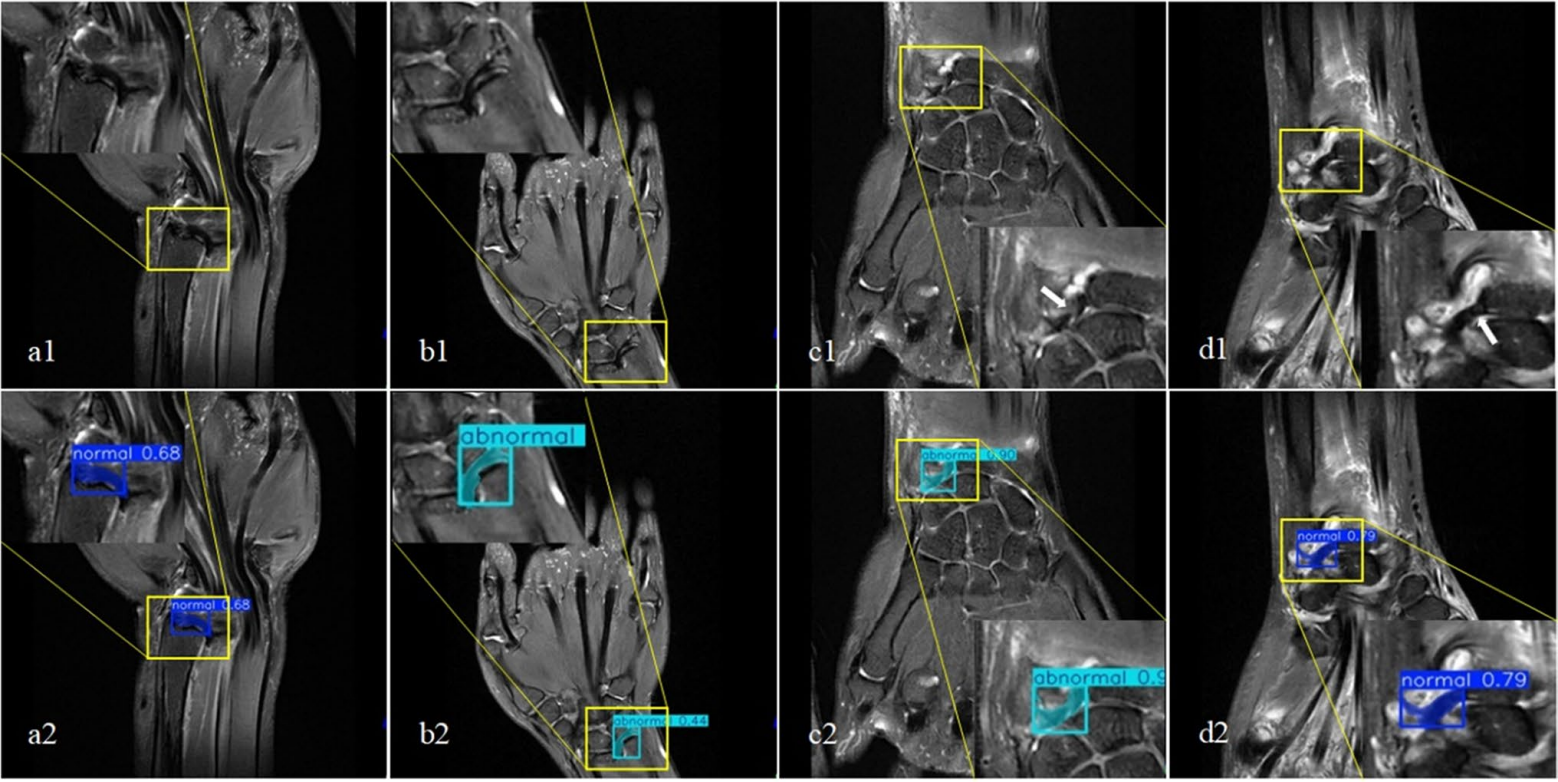
Example of Yolo11l model prediction results. A layer with a normal central structure (a1) was predicted to obtain the correct normal result (a2), which is a true negative (TN) result. A layer with normal peripheral structure (b1) was predicted to have an erroneous abnormal result (b2), namely a false positive (FP) result. The layer (c1) with signal discontinuity (white arrow) in another peripheral structure was predicted to obtain the correct abnormal result (c2), which is the true positive (TN) result. Finally, a layer with slightly higher signal injury (white arrow) was observed within the peripheral structure (c1), and after prediction, an incorrect normal result (c2) was obtained, which is a false negative (FN) result

In the analysis, the YOLO11l model achieved better diagnostic performance with sensitivity of 79.0% (71.0% ~ 87.0%), specificity of 70.0% (61.0% ~ 79.0%), and accuracy of 74.5% (68.5% ~ 80.5%). These metrics were non-inferior to those of Resident 1 (sensitivity: 74.0% (65.4% ~ 82.6%); specificity: 64.0% (54.6% ~ 73.4%); accuracy:69.0% (62.6% ~ 75.4%)). Attending 2 demonstrated the highest sensitivity (87.0% (80.4% ~ 93.6%)), specificity (80.0% (72.8% ~ 87.2%)), and accuracy (83.5% (78.3% ~ 88.7%)). There was no statistical difference in diagnostic performance between Resident 1 and this model (p = 0.41), but a significant statistical difference was found compared with Attending 2 (p = 0.015). Figure 5 shows examples of different recognition results, including true positive, true negative, false positive, and false negative.

## Discussion

Our study advances TFCC structural diagnosis in three key areas: first, the YOLO segmentation model provides an important foundation for accurate anatomical depiction, which is particularly important for TFCC detection. Second, the YOLO11l model, combined with AM, exhibits enhanced performance, with improved detection rate and diagnostic accuracy. Third, the model offers notable advantages in processing speed, thereby boosting its clinical utility. Notably, its diagnostic performance is non-inferior to radiologists with one year of general diagnostic experience but no specialized training in MSK imaging, supporting radiologists as a valuable auxiliary diagnostic tool.

Accurate diagnosis of TFCC injuries remains clinical challenge, especially for peripheral subtle lesions. A prior study reported suboptimal diagnostic performance among radiology residents when evaluating the dorsal and volar distal radioulnar ligaments in 2D MRI (9). AI has demonstrated scalability and efficiency in automating repetitive tasks, enabling clinicians and radiologists to focus on complex aspects of patient care (26–28), which capability particularly valuable for peripheral TFCC diagnosis where detection is critical. The YOLO architecture has demonstrated performance in MSK imaging research: the YOLOv8 model achieved effective detection and classification of lumbar disc herniation with high concordance to expert radiologist classifications (29). Another study validated the superior diagnostic capacity of YOLO models for temporomandibular joint disc assessment, which significantly improved the diagnosis of articular disorder (30). Recent studies have highlighted the potential of YOLOv8 for detecting early-stage osteonecrosis (31), which aligns with our use of a single-sequence MRI approach to enable comprehensive TFCC evaluation. A previous study utilized algorithm learning to evaluate and classify the features of contrast agent regions, rather than learning complete MRI scans with TFCC lesions (32), while our study directly conducted semantic learning at the 2D FS-T2WI level. This distinction underscores our approach focus on full structural assessment rather than narrow feature subsets, which better aligns with clinical needs for comprehensive TFCC injury detection. However, in contrast to these prior findings, our study shows relatively low diagnostic efficacy for peripheral injuries (Table 4), primarily reflected in specificity. One potential explanation is that the complexity and heterogeneity of peripheral structural injuries make full visualization at the coronal plane challenging. Another contributing factor is the limited dataset size and insufficient effective semantic information to distinguish normal from pathological structures. This indicates that future work should prioritize addressing these limitations. But the model retains a high true positive rate, which is a key strength. These findings suggest that AI-based models, such as YOLO11l, can effectively supplement and enhance the ability of medical professionals to detect TFCC structural injuries.

Our YOLO model employs semantic segmentation to achieve precise structural depiction, improving diagnostic accuracy through the following mechanisms: (1) accurate differentiation between TFCC and surrounding tissues (reducing misclassification), (2) detailed delineation of the bone-TFCC interface, and (3) complete morphological characterization (including position, shape, and size) to support clinical decision-making. Although its Dice coefficient is not perfect, it still captures the vast majority of key structures within each slice, which is crucial for ensuring the accuracy of final detection outcomes.

The integration of AM allows the model to selectively focus on diagnostically relevant image regions, enhancing model performance while optimizing computational and diagnostic efficiency. This approach has been clinically validated in medical imaging applications (33–35). YOLOv11 innovatively incorporates a novel Cross Stage Partial with Spatial Attention (C2PSA) block (36), which significantly enhances spatial attention in feature maps. This allows the model to prioritize diagnostically relevant regions, a feature particularly beneficial for detecting small or partially occluded anatomical structures and thus improving its object detection and analysis capabilities. This enhanced spatial awareness contributes to improve the overall performance of YOLO11, particularly in visually complex imaging scenarios (12). While this method has not been investigated in MSK radiology, its efficacy has been validated in other medical imaging domains, specifically for the automated detection of exudate in retinal fundus images (37). In our external test set, YOLOv11 models showed modest performance improvements compared to their YOLOv8 counterparts of the same size, which also supports the potential of AM for medical diagnostic applications.

Our findings highlight the substantial diagnostic potential of the YOLO11l model for the pathological evaluation of TFCC. When evaluating some structural lesions of TFCC on a sampled dataset, the model’s diagnostic performance is non-inferior to that of radiology resident 1 (p = 0.41), confirming that the model is non-inferior to radiologists with one year of general diagnostic experience but no specialized training in MSK imaging. However, its performance is significantly inferior to that of Attending 2 (p = 0.02), indicating that radiologists with extensive clinical experience achieve superior diagnostic outcomes. These results underscore the model’s value as a reliable auxiliary tool for junior radiology residents. A typical clinical scenario involves Resident 1 misinterpreting subtle signal changes on FS-T2WI images as normal. Even experienced radiologists may find diagnosing TFCC ulnar attachment tears challenging. This emphasizes the potential of the model as a reliable tool for complex imaging studies such as MRI. Furthermore, the inherent subjectivity of MRI interpretation may lead to diagnostic variability both between radiologists and within individual radiologists' evaluations over time. For instance, a radiologist may identify subtle lesions during an initial assessment, but miss them in subsequent reviews due to inconsistent interpretation criteria or cognitive fatigue. The model mitigates these inconsistencies by delivering standardized, objective assessments, thereby ensuring more reliable and reproducible diagnoses. These findings confirm that AI-based models can effectively supplement and strengthen the ability of medical professionals to detect TFCC structural injuries.

While these findings are promising, this study has several limitations that warrant acknowledgment. First, the sample size may be insufficient, which have prevented the model from achieving its optimal performance. Second, the potential synergistic benefits of combining multi-planar MRI sequence remain unexamined, representing opportunities for further performance optimization. Third, the YOLO11l model only demonstrates slightly better diagnostic performance relative to residents with limited MSK subspecialty experience, leaving room for algorithmic optimization to further improve its diagnostic performance. Finally, subgroup analyses assessing potential model biases (e.g., age, gender, or group of lesion areas) were not conducted. Notably, significant age differences existed between our injury and healthy control group. In future research, we will include more samples, improve algorithms, and combine demographic and clinical variables to better characterize model performance across subgroups of populations.

In conclusion, our study preliminarily validates the efficacy of YOLO-based models for diagnosing TFCC structural injuries. The diagnostic improvement achieved by the YOLOv11 architecture with AM demonstrates the potential of AM in improving the accuracy of medical image analysis (38). Future research should explore the wider application of AI in medical imaging tasks, and investigate complementary techniques to further optimize performance.

## Supplementary Information

Below is the link to the electronic supplementary material.

Supplementary file1 (DOCX 1.04 MB)

## Data Availability

The data that support the fndings of this study is available on request from the corresponding author. The data are not publicly available due to privacy or ethical restrictions.

## Abbreviations

TFCC: Triangular Fibrocartilage Complex
FS-T2WI: Fat-Suppressed T2-Weighted Images
PACS: Picture Archiving and Communication System
AM: Attention Mechanisms
PSA: Pyramid Squeeze Attention
YOLO: You Only Look Once
