# Supplementary material for: Identification of TFCC substructure injury in wrist MRI using computer vision: a diagnostic aid for radiologists
Source: Skeletal Radiol. 2025 Dec 19;55(5):1045–57. doi: 10.1007/s00256-025-05106-x (PMC13018085; doi:10.1007/s00256-025-05106-x)
Supplement: Supplementary file 1 — Supplementary file1 (DOCX 1.04 MB) [file 256_2025_5106_MOESM1_ESM.docx]

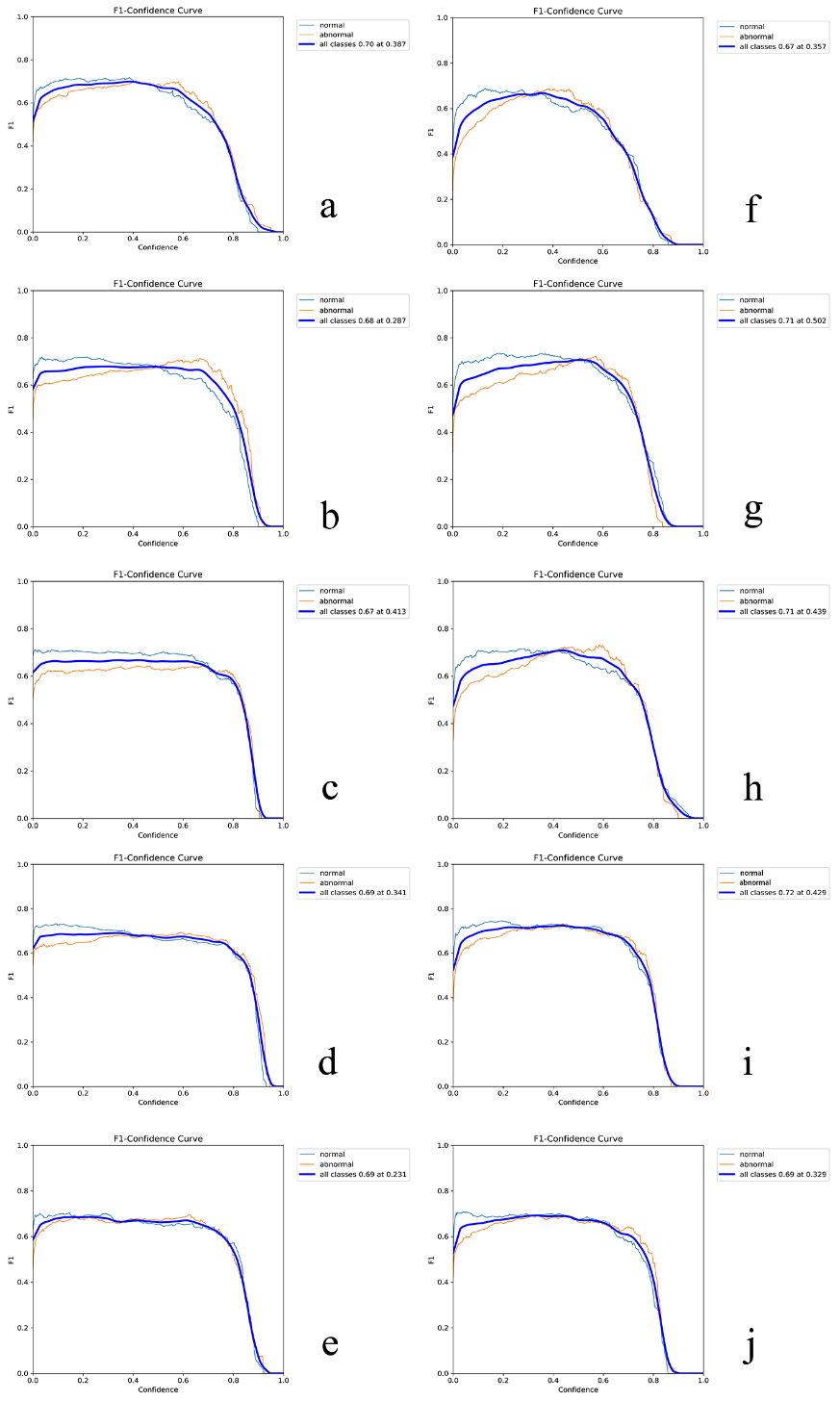


**Supplementary Figure 1.** Comparison of classification performance F1-Confidence curve of 10 types of YOLO models after training and validation. Respectively represent the YOLOv8n-seg (a), YOLOv8s-seg (b), YOLOv8m-seg (c), YOLOv8l-seg (d), YOLOv8x-seg (e), YOLO11n-seg (f), YOLO11s-seg (g), YOLO11m-seg (h), YOLO11l-seg (i), and YOLO11x-seg (j).


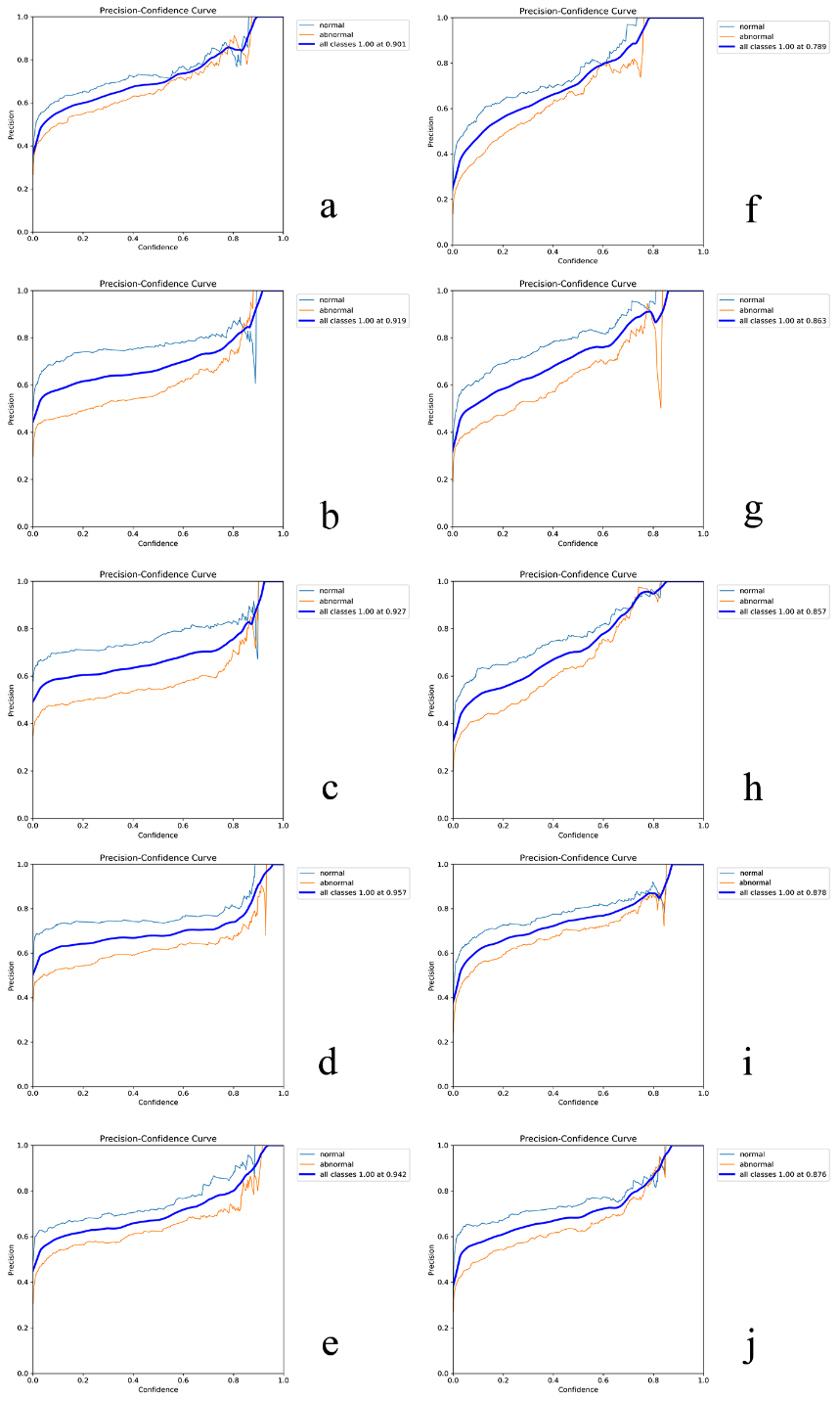


**Supplementary Figure 2.** Comparison of classification performance Precision-Confidence curve of 10 types of YOLO models after training and validation. Respectively represent the YOLOv8n-seg (a), YOLOv8s-seg (b), YOLOv8m-seg (c), YOLOv8l-seg (d), YOLOv8x-seg (e), YOLO11n-seg (f), YOLO11s-seg (g), YOLO11m-seg (h), YOLO11l-seg (i), and YOLO11x-seg (j).


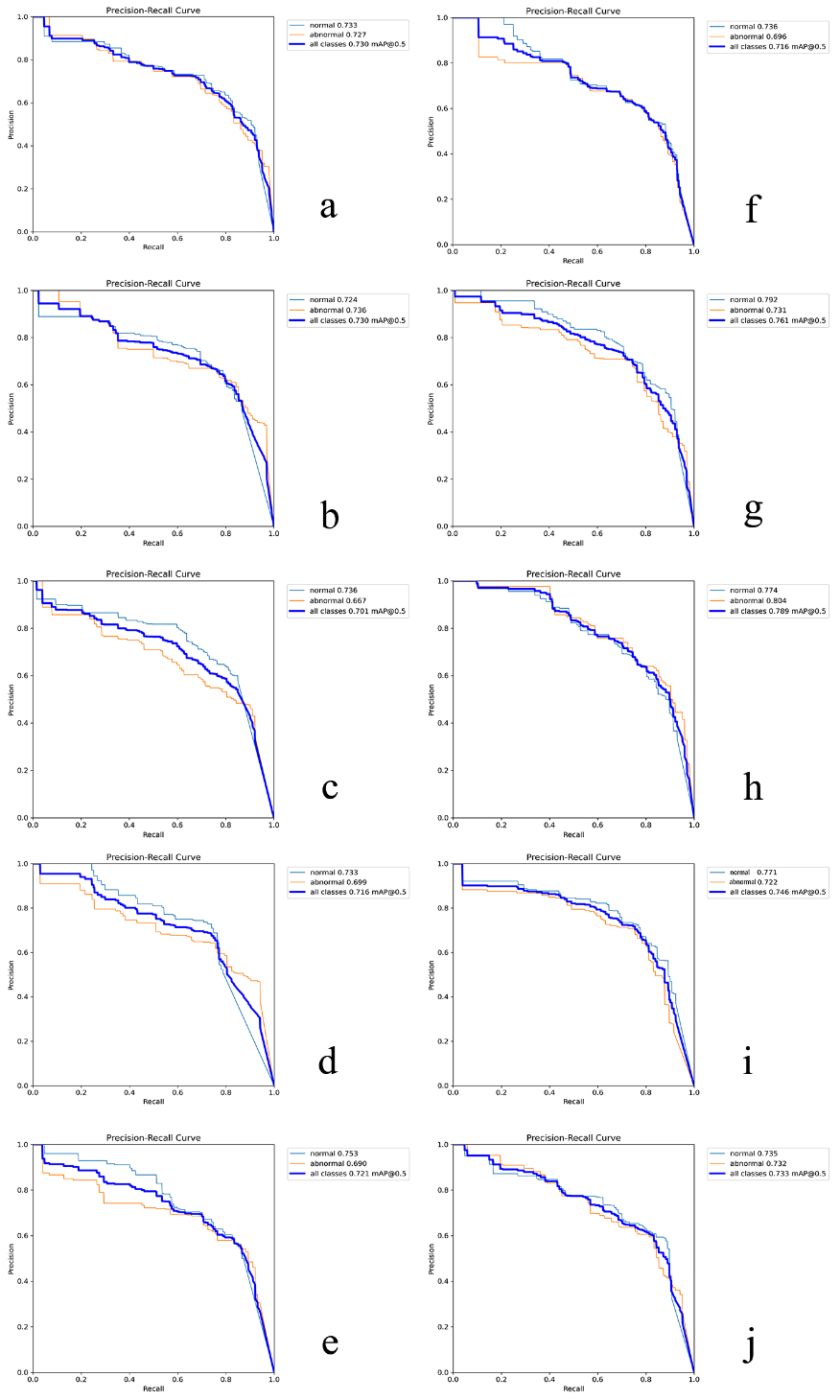


**Supplementary Figure 3.** Comparison of classification performance Precision-Recall curve of 10 types of YOLO models after training and validation. Respectively represent the YOLOv8n-seg (a), YOLOv8s-seg (b), YOLOv8m-seg (c), YOLOv8l-seg (d), YOLOv8x-seg (e), YOLO11n-seg (f), YOLO11s-seg (g), YOLO11m-seg (h), YOLO11l-seg (i), and YOLO11x-seg (j).
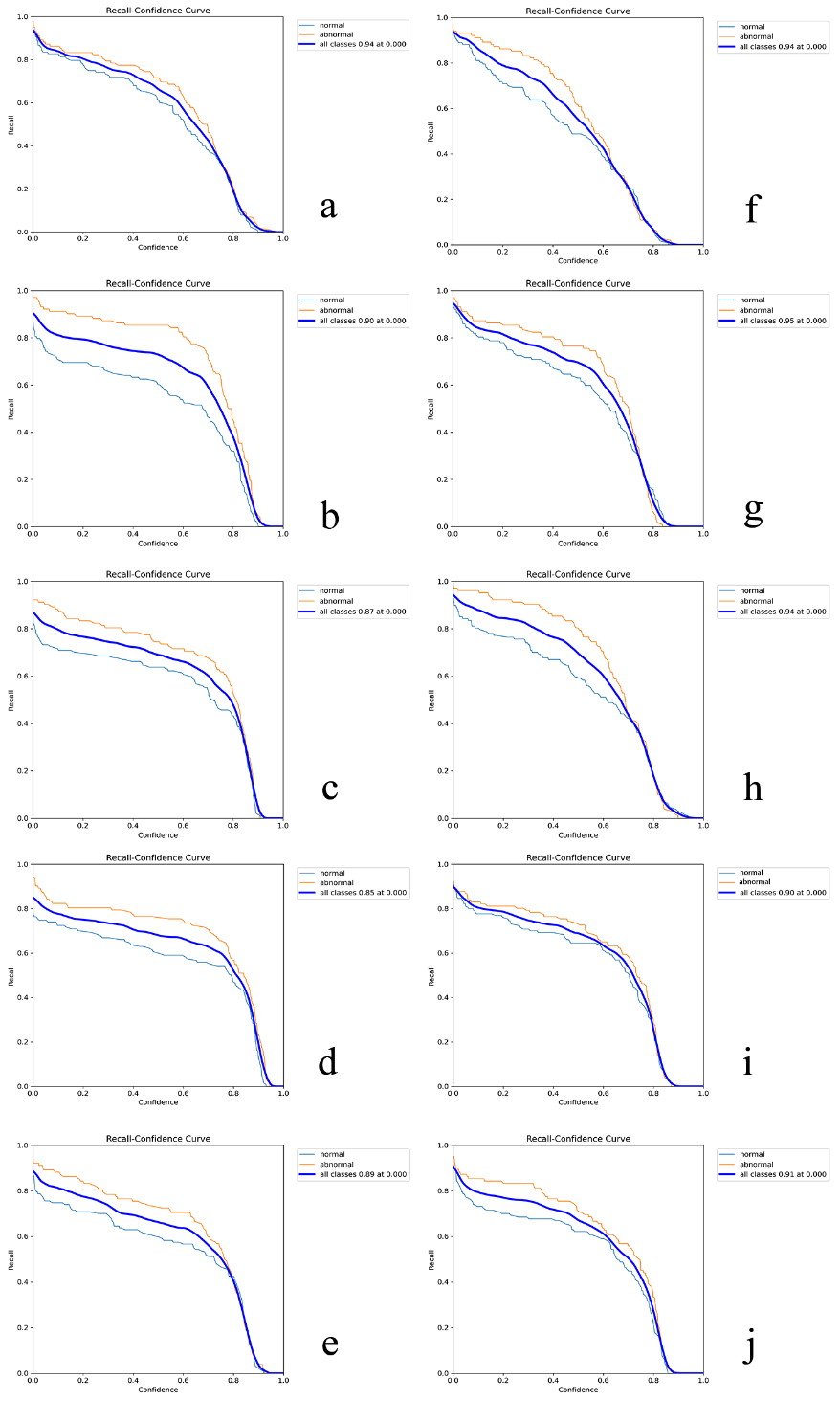


**Supplementary Figure 4.** Comparison of classification performance Recall-Confidence curve of 10 types of YOLO models after training and validation. Respectively represent the YOLOv8n-seg (a), YOLOv8s-seg (b), YOLOv8m-seg (c), YOLOv8l-seg (d), YOLOv8x-seg (e), YOLO11n-seg (f), YOLO11s-seg (g), YOLO11m-seg (h), YOLO11l-seg (i), and YOLO11x-seg (j).

| **Supplementary Table 1. The segmentation performance of the model in both internal and external test sets** | | | | |
| --- | --- | --- | --- | --- |
| model | Internal test set | p | External test set | p |
| YOLOv8n | 0.804803021 |  | 0.646214219 | ＜0.00001^!^ |
| YOLOv8s | 0.807808317 |  | 0.712203977 | 0.00096^!^ |
| YOLOv8m | 0.796784849 | 0.00487121^#^ | 0.615708378 | 0.00008^!^ |
| YOLOv8l | 0.803082431 |  | 0.659332504 | ＜0.00001^!^ |
| YOLOv8x | 0.791745963 |  | 0.680828278 | ＜0.00001^!^ |
| YOLO11n | 0.798084292 |  | 0.649758572 | ＜0.00001^!^ |
| YOLO11s | 0.80324532 |  | 0.72786427 | ＜0.00001^!^ |
| YOLO11m | 0.802325605 |  | 0.720924268 | ＜0.00001^!^、0.00141* |
| YOLO11l | 0.817614167 |  | 0.768832363 | ＜0.00001^!^ |
| YOLO11x | 0.787262282 |  | 0.672870725 |  |
| #There is a statistical difference between 8n vs 8m, as indicated by the Wilcoxon signed-rank test with Bonferroni correction (p<0.005)  ! There are statistical differences between 8n and 8s, 8n and 8m, 8s and 8m, 8m and 8l, 8m and 8x, 11n and 11s, 11n and 11m,11s and 11l, 11m and 11l, and Wilcoxon signed rank test with Bonferroni correction (p<0.005)  *There is a statistical difference between 8m and 11m, as tested by Wilcoxon signed rank test (p<0.05) | | | | |

| **Supplementary Table 2. Regional classification diagnostic Performance of YOLOv8 and YOLO11 Models on Testing Datasets** | | | | | | | | | | | | |
| --- | --- | --- | --- | --- | --- | --- | --- | --- | --- | --- | --- | --- |
|  | Internal Testing Dataset | | | | | | External Testing Dataset | | | | | |
|  | Sensitivity | | Specificity | | Accuracy | | Sensitivity | | Specificity | | Accuracy | |
| Partition  Model | central | peripheral | central | peripheral | central | peripheral | central | peripheral | central | peripheral | central | peripheral |
| YOLOv8n | 91.67% (83.85% - 99.49%) | 83.33% (72.79% - 93.88%) | 63.33% (46.09% - 80.58%) | 80.72% (72.24% - 89.21%) | 80.77% (72.02% - 89.52%) | 81.68% (75.05% - 88.30%) | 65.57% (53.65% - 77.50%) | 49.21% (36.86% - 61.55%) | 74.19% (58.79% - 89.60%) | 61.21% (53.78% - 68.65%) | 68.48% (58.98% - 77.97%) | 57.89% (51.49% - 64.30%) |
| YOLOv8s | 91.67% (83.85% - 99.49%) | 81.25% (70.21% - 92.29%) | 83.33% (70.00% - 96.67%) | 72.29% (62.66% - 81.92%) | 88.46% (81.37% - 95.55%) | 75.57% (68.21% - 82.93%) | 73.77% (62.73% - 84.81%) | 60.32% (48.24% - 72.40%) | 70.97% (54.99% - 86.95%) | 61.21% (53.78% - 68.65%) | 72.83% (63.74% - 81.92%) | 60.96% (54.63% - 67.30%) |
| YOLOv8m | 95.83% (90.18% - 101.49%) | 81.25% (70.21% - 92.29%) | 86.67% (74.50% - 98.83%) | 73.49% (64.00% - 82.99%) | 92.31% (86.39% - 98.22%) | 76.34% (69.06% - 83.61%) | 77.05% (66.50% - 87.60%) | 80.95% (71.26% - 90.65%) | 61.29% (44.14% - 78.44%) | 58.79% (51.28% - 66.30%) | 71.74% (62.54% - 80.94%) | 64.91% (58.72% - 71.11%) |
| YOLOv8l | 91.67% (83.85% - 99.49%) | 75.00% (62.75% - 87.25%) | 83.33% (70.00% - 96.67%) | 77.11% (68.07% - 86.15%) | 88.46% (81.37% - 95.55%) | 76.34% (69.06% - 83.61%) | 83.61% (74.32% - 92.90%) | 66.67% (55.03% - 78.31%) | 80.65% (66.74% - 94.55%) | 53.94% (46.33% - 61.54%) | 82.61% (74.86% - 90.35%) | 57.46% (51.04% - 63.87%) |
| YOLOv8x | 83.33% (72.79% - 93.88%) | 70.83% (57.97% - 83.69%) | 80.00% (65.69% - 94.31%) | 75.90% (66.70% - 85.10%) | 82.05% (73.53% - 90.57%) | 74.05% (66.54% - 81.55%) | 78.69% (68.41% - 88.97%) | 68.25% (56.76% - 79.75%) | 70.97% (54.99% - 86.95%) | 46.67% (39.05% - 54.28%) | 76.09% (67.37% - 84.80%) | 52.63% (46.15% - 59.11%) |
| YOLO11n | 95.83% (90.18% - 101.49%) | 77.08% (65.19% - 88.97%) | 83.33% (70.00% - 96.67%) | 74.70% (65.35% - 84.05%) | 91.03% (84.68% - 97.37%) | 75.57% (68.21% - 82.93%) | 63.93% (51.88% - 75.98%) | 55.56% (43.29% - 67.83%) | 77.42% (62.70% - 92.14%) | 55.76% (48.18% - 63.34%) | 68.48% (58.98% - 77.97%) | 55.70% (49.25% - 62.15%) |
| YOLO11s | 97.92% (93.88% - 101.96%) | 75.00% (62.75% - 87.25%) | 83.33% (70.00% - 96.67%) | 77.11% (68.07% - 86.15%) | 92.31% (86.39% - 98.22%) | 76.34% (69.06% - 83.61%) | 70.49% (59.05% - 81.94%) | 63.49% (51.60% - 75.38%) | 77.42% (62.70% - 92.14%) | 63.03% (55.66% - 70.40%) | 72.83% (63.74% - 81.92%) | 63.16% (56.90% - 69.42%) |
| YOLO11m | 93.75% (86.90% - 100.60%) | 81.25% (70.21% - 92.29%) | 80.00% (65.69% - 94.31%) | 73.49% (64.00% - 82.99%) | 88.46% (81.37% - 95.55%) | 76.34% (69.06% - 83.61%) | 91.80% (84.92% - 98.69%) | 87.30% (79.08% - 95.52%) | 64.52% (47.67% - 81.36%) | 47.88% (40.26% - 55.50%) | 82.61% (74.86% - 90.35%) | 58.77% (52.38% - 65.16%) |
| YOLO11l | 91.67% (83.85% - 99.49%) | 91.67% (83.85% - 99.49%) | 83.33% (70.00% - 96.67%) | 73.49% (64.00% - 82.99%) | 88.46% (81.37% - 95.55%) | 80.15% (73.32% - 86.98%) | 91.80% (84.92% - 98.69%) | 77.78% (67.51% - 88.04%) | 90.32% (79.92% - 100.73%) | 55.76% (48.18% - 63.34%) | 91.30% (85.55% - 97.06%) | 61.84% (55.54% - 68.15%) |
| YOLO11x | 85.42% (75.43% - 95.40%) | 83.33% (72.79% - 93.88%) | 80.00% (65.69% - 94.31%) | 78.31% (69.45% - 87.18%) | 83.33% (75.06% - 91.60%) | 80.15% (73.32% - 86.98%) | 78.69% (68.41% - 88.97%) | 73.02% (62.06% - 83.98%) | 77.42% (62.70% - 92.14%) | 49.09% (41.46% - 56.72%) | 78.26% (69.83% - 86.69%) | 55.70% (49.25% - 62.15%) |
